# Induction of Hyperalgesia in Pigs through Blocking Low Hydraulic Resistance Channels and Reduction of the Resistance through Acupuncture: A Mechanism of Action of Acupuncture

**DOI:** 10.1155/2013/654645

**Published:** 2013-08-13

**Authors:** Wei-Bo Zhang, Yi-Hui Xu, Yu-Ying Tian, Hong Li, Guang-Jun Wang, Tao Huang, Shu-Yong Jia

**Affiliations:** ^1^Institute of Acupuncture & Moxibustion, China Academy of Chinese Medical Sciences, Beijing 100700, China; ^2^Department of Geriatrics, First People's Hospital, Kunshan 215300, China

## Abstract

According to the classic theory of Chinese medicine, pain is due to the blockage in meridian channels, and acupuncture was invented to treat pain by “dredging” the channels. To test the theory, a hyperalgesia model was made by injecting hydrogel into low hydraulic resistance channel (LHRC) in 12 anaesthetized minipigs. Tail-flick threshold and ear-flick threshold were measured using a thermal radiation dolorimeter, and relative flick threshold (RFT) was calculated. Hydraulic resistance (HR) was measured with a biological HR measuring instrument on low HR points on LHRC and on control points with higher HR located outside LHRC; readings were recorded before, during, and after acupuncture treatment. RFT decreased after blocking the LRHC and was still significantly decreased 2 days and 4 days afterwards. No significant changes occurred when injecting saline into the same points or injecting gel into points outside the channel. Subsequent acupuncture reduced HR on LRHC along meridians but had no significant effect on sites with higher HR located outside LHRC. One of the mechanisms of action of acupuncture treatment for chronic pain may be that acupuncture affects peripheral tissue by reducing the HR in LHRC along meridians, improving the flow of interstitial fluid and removing algogenic substances and thereby relieving pain.

## 1. Introduction and Background

### 1.1. The Concept of Pain and Its Treatment Based on Meridian Channels in the Han Dynasty Classic, The Yellow Emperor's Inner Canon

According to *The Yellow Emperor's Inner Canon*, compiled between 206 BCE and 220 CE, there are meridians and collaterals in the human body constituting a network of channels through which *qi* and *blood* flow. Diseases, such as pain, are caused by *qi* stagnation or *blood* stasis of the channels which slows down the flow of *qi* and *blood*. Acupuncture was invented to treat diseases by inserting a fine needle into the skin at designated points (acupuncture points) and often replaced herbal medicine or puncturing and scraping with *Bian*-stones. Acupuncture regulates *qi* and *blood*, promotes the circulation of *qi* and *blood*, and harmonizes and adjusts healthy and pathogenic *qi* in the channels [1]. How should we understand this theory? Is it actually describing reality or is it just a metaphor? A recent meta-analysis showed that acupuncture can effectively relieve chronic pain [2]. If such curative effects could be adequately explained by modern medical science, we would not need to consider the existence of meridian channels. Let us consider some of these possible explanations. 

Neural mechanisms are a popular explanation for why acupuncture seems to be effective for treating pain. Zhang put forward a hypothesis based on neural interactive mechanisms in the thalamus to explain the pain treatment [4]. Han found that the role of endorphins was a key in acupuncture analgesia [3]. However, neither of these neural mechanisms can sufficiently explain some features of chronic pain and its treatment [5]. 

It is now relatively well accepted that the treatment of pain with acupuncture can be explained through gate control theory, as developed by Melzack and Wall in 1965 [6]. According to this theory, acupuncture signals are transmitted through types II and III nerve fibers, which accounts for the sensations of aching, numbness, distension, and heaviness reported by patients. The signals can inhibit the input of pain signals which are primarily transmitted through type IV nerve fibers (A_*δ*_ and C fibers specifically). This explanation works well when applied to acupuncture anaesthesia during surgery, but it is not sufficient to explain why chronic pain can be inhibited not only during an acupuncture session, but also for hours and days afterwards, when gate control signals clearly no longer exist. The meta-analyses by Hopton and MacPherson showed that real acupuncture is significantly more effective for knee pain and tension-type headache in terms of long-term outcomes (6 to 12 months) compared to sham acupuncture [2]. The fact that acupuncture at points distal to the site of pain has also been shown to effectively treat chronic pain, such as heel pain [7], trigeminal neuralgia [8], and scapulohumeral periarthritis [9], is further evidence of the inadequacy of gate control theory as an explanation.

Acupuncture sensation (Deqi) is quite important for treating some conditions, but is not necessary for all acupuncture therapies. For example, shallow acupuncture, in which the stimulation is very light and does not produce a Deqi effect, has been shown to be no less effective than deep acupuncture for some diseases [10]. In meridian-sinew therapy, a needle knife is used to separate tissue adhesions to relieve pain. During this procedure local anaesthesia is applied by injecting a small amount of lidocaine into the operating site, thereby blocking any neural signals which might be involved in gate control. Studies have shown that the release of adhesion with the needle knife under local anesthesia has positive effects on stiffness of the knee, motor functions, and pain control [11], none of which can be explained through gate control theory. Another example of “light sensation” acupuncture is abdomen acupuncture which has been shown to have similar rates of effectiveness for treating cervical spondylosis compared with electrical acupuncture [12]. In addition, moxibustion and massage on the meridian channels do not strongly produce Deqi sensations, and yet they still have curative effects. It has been noted that acupuncture is not only a treatment for pain but can also treat or improve many other kinds of pathological conditions. Pain is usually a signal to alert us to a pathological process; therefore, merely inhibiting the pain without treating the underlying condition is not beneficial to our overall health. Therefore acupuncture should not be regarded as merely a unique method of pain inhibition based on the interaction between nerve signals. 

Huang, a senior philologist in China, has described acupuncture points as analogous to turning on a switch [13]. According to the* Yellow Emperor's Inner Canon*, acupuncture exerts its effects by opening meridian channels like a switch. Understanding the nature of the meridian channels is essential for carrying out valid, relevant research on acupuncture.

### 1.2. Three Stages of Pain Formation and Related Treatments

At this point, we still do not fully understand how and why acupuncture can effectively treat chronic pain. One of the main reasons is that the phenomena of pain and the consciousness of pain are very complicated. Pain involves at least three stages: the stimulating source of pain, the transmission of pain signals, and the processing of pain signals in the central nervous system. Gate control is a process of inhibiting pain signals at the third stage, but may also come into play when giving an intervention at the first or second stage. In western medicine, severe pain that does not respond to other treatments may be treated by damaging the afferent nerves to prevent the input of pain signals. However, since pain is an alarm signal indicating a pathological state in the organs or tissue, blocking the input of pain signals or changing the processing of pain signals in the central nerve system can only temporally relieve symptoms. It will not resolve the problem in the organs or tissues, especially since the most common sources of pain are inflammation, tumors, or tissue injuries, which cannot be addressed through the nervous system alone. 

### 1.3. Biochemicals Produced by Inflammation Are the Primary Causative Factor for Chronic Pain

In pain is physiology, pain often related to the process of inflammation, during which many chemicals like bradykinin (BK), prostaglandin (PG), 5-HT, substance P (SP), histamine, and so forth are released from the inflammatory site, leading to hyperalgesia and allodynia [14]. Pressing, stretching, or moving the affected area often causes additional pain, often limiting the movements of chronic pain patients. The other important factor in chronic pain is the accumulation of metabolites, leading to excess H^+^ and causing hyperalgesia or pain. This happens when someone exercises too much and lactic acid is produced too quickly to be cleaned up by the circulation system. Logically, if the chemicals which induce the pain are eliminated, the pain will be permanently relieved and we can call this “curing the pain” instead of “analgesia.” We can draw parallels with aspirin therapy in western medicine, which is used to inhibit the enzymes that make prostaglandins (PG). Acupuncture may treat pain in a similar fashion by cleaning up algogenic substances. 

In the theory of TCM, meridian channels are the channels in which *qi* and *blood* flow; therefore if blockage occurs, pain will result. This is quite similar to the processes mentioned earlier. Our hypothesis is that pain or hyperalgesia is caused by meridian channel stasis, leading to an accumulation of metabolites and inflammatory substances in local tissue. Acupuncture works by cleaning up the algogenic substances in local tissue through dredging the meridian channels. 

The first author of this paper, Zhang, has recently shown that one important aspect of the physical basis of the meridian channels is low hydraulic resistance channel (LHRC) that exists along meridians and facilitates the flow of interstitial fluid according to Darcy's law [15]. If the channel is blocked, the interstitial flow will be partially interrupted and this, in turn, will cause an accumulation of metabolic and inflammatory substances. Is it possible to induce hyperalgesia or pain by blocking these LHRC? Can acupuncture open the channels by diminishing the stasis, specifically lowering the hydraulic resistance? Two experiments were designed to answer the questions.

## 2. Materials and Methods

### 2.1. A Hyperalgesia Model Induced by Injecting Hydrogel into LHRC

#### 2.1.1. Animal Preparation

The experiment was carried out on twelve anaesthetized Chinese minipigs (9~11 kg, males, one month of age) which were provided by the Animal Center of Beijing Agriculture University and were kept according to the guidelines issued by Beijing Municipal Administrative Committee for experimental animals. The pigs were forbidden to eat before the operation and were anaesthetized by injecting 1.5 to 2 mg/kg of 2% phenobarbital sodium solution intraperitoneally. After the experiment, the pigs were sent back to the Animal Center to recover for several days until the next experiment.

#### 2.1.2. Measurement of Meridian Line and Low Hydraulic Resistance Point

**Figure 1:**
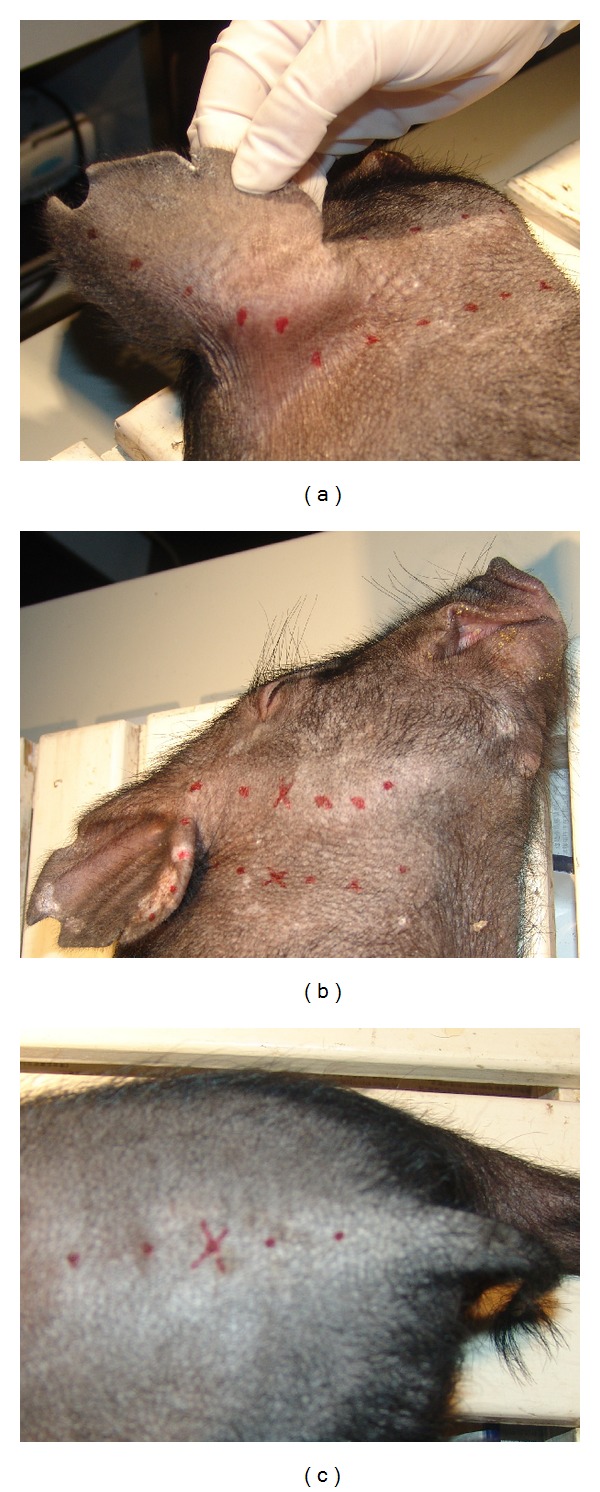
(a) A low impedance meridian line propagated along the neck, back of the ear, and the tip of ear (represented by red dots). (b) A low impedance meridian line propagated along the lower jaw and extended to the helix. (c) A low impedance meridian line propagated along the midline of the back and extended to the tail. (× marked the position of low hydraulic resistance points where hydrogel was injected).

The meridian lines which pass through the ears and tail were measured with an electric impedance meridian locator (type: 57-6F30, made in Donghua factory in China). Two meridian lines were found passing across the ear. One extended from the tip of the ear, down the back of the ear, and across the neck (Figure 1(a)), following a trajectory similar to the gallbladder meridian. The other went from the helix on the ear to the lower jaw (Figure 1(b)), making it similar to the pathway of the stomach meridian. Another meridian line was found along the midline of pig's back and extended down to the tail (Figure 1(c)), similar to the Governor (*Du*) meridian. The tip of the ear, the helix, and the middle of the tail were chosen to measure pain thresholds.

**Figure 2:**
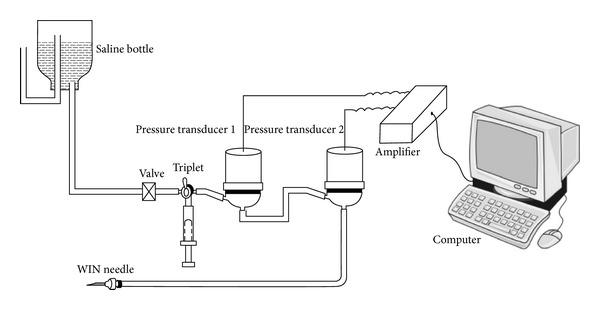
The system of measuring hydraulic resistance on subcutaneous tissue in pigs [3].

Low hydraulic resistance points (LHRP) were located by using a biological hydraulic resistance measuring instrument with two pressure transducers in a row (Figure 2, made in Zhang's lab in China) on areas 7~9 cm from the low impedance meridian lines (Figures 1(b) and 1(c)). The principle of measuring LHRP and the calculation of hydraulic resistance was introduced in detail in a previous paper [16].

#### 2.1.3. Preparation and Injection of Hydrogel

Polyacrylamide hydrogel was obtained from *Jilin Aodong* Biomaterial Company in China. One gram of pure Polyacrylamide hydrogel was diluted by mixing 5 mL 0.9% saline in order to make it suitable for injection. The 12 pigs were randomly divided into three groups, with four pigs in each group. In the experimental group, after a LHRP was found, the needle was fixed on the skin with glue and 0.5 mL polyacrylamide hydrogel was slowly injected into the LHRP through the measured needle to block the channel. In the first control group, saline was injected instead of hydrogel but in the same amount and at the same positions. In the second control group, hydrogel was injected at high hydraulic resistance points (HHRP) located 2-3 cm away from LHRC. 

#### 2.1.4. Measurement of Pain Threshold and the Procedure

A thermal radiation dolorimeter (made by the Institute of Acupuncture in China) with a 0.38 cm^2^ hot point and fixed distance of 0.5 cm to the skin was used to measure the pain threshold which was represented by ear-flick threshold and tail-flick threshold, respectively. The instrument has been used in previous studies to measure the tail-flick threshold in rats [17]. The device was never used for more than 15 s in order to prevent tissue damage. The flick thresholds (FT) on the ear tips, the helix on both sides, and the middle of tail, a total of five places, were measured by counting the time between the start of radiation and the flick of the ears or tail with a stopwatch. FT on each place was measured three times with three-minute intervals between the measurements and the average FT was calculated. For each pig, FT was measured before the injection, immediately after the injection, and two days and four days after the injection, respectively. A reference point, at which no injections were given during the time of the experiment, was chosen to eliminate the influence of different anesthesia levels on FT. If FT on the ear tip or helix was used as the site of measurement during the experiment, the middle of the tail was used as a reference. Conversely, if the middle of the tail was used as the site of measurement during the experiment, the ear tip was measured as the reference point. 

#### 2.1.5. Statistics

The ratio between experimental FT and reference FT was calculated, representing the relative changes of FT (RFT) at the point where the channel was blocked at a distance along the meridian. For each pig, five positions, left ear tip, helix, right ear tip, helix, and tail, were measured. There were 20 measurements in total on the four pigs in each group. One-way ANOVA variance analysis was used to examine the significance of mean RFT following the experiment, and Wilcoxon Signed Ranks Test with 2-tailed analysis was used to examine the mean RFT between the two time points. The level of statistical significance was set at 0.05 for all analyses. Statistical analysis was performed using SPSS 13.0 software.

### 2.2. Observing the Change in Hydraulic Resistance along the Channel When Acupuncture Is Performed on Pigs

**Figure 3:**
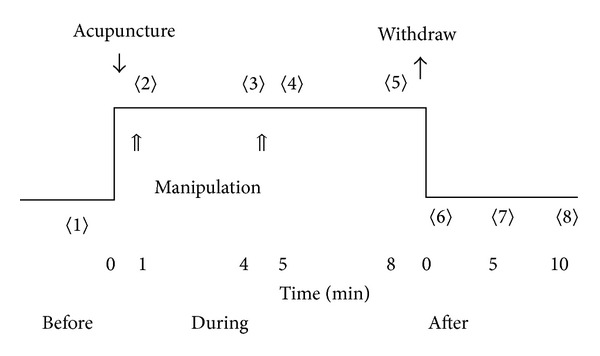
Measurement profile of the hydraulic resistance in acupuncture performed on pigs.

The experiment was carried out on three anaesthetized minipigs (9~12 kg, males). The animal preparation was the same as in the experiment mentioned in Section 2.1.1. Thirteen meridian lines which were similar to the stomach meridian and gallbladder meridian were measured by an electric impedance meridian locator (type: 57-6F30, made in *Donghua* Electric Instrument Factory in China). The LHRPs were located using a biological hydraulic resistance measuring instrument (Figure 2) along the low impedance meridian lines. After a LHRP was found, the measuring needle was fixed by glue on the skin for continuous measurement. A point along the meridian line 4~6 cm away from the LHRP was chosen for acupuncture needling. After inserting the acupuncture needle into the skin, manipulation using lift-thrusting and twirling the needle was performed for about one minute, and the needles were retained for 3 minutes. Manipulation was then performed again for one minute, the needles were retained for 3 more minutes, and then the needle was withdrawn. HR was measured before the acupuncture (1), after the first manipulation (2), 3 minutes later (3), after the second manipulation (4), 3 minutes later (5), after withdrawing the needle (6), 5 minutes later, (7) and 10 minutes later (8) (Figure 3). Each measurement lasted 6 seconds. For one meridian line, a LHRP and a higher hydraulic resistance point (HHRP) 1-2 cm lateral to the LHRP were measured randomly with the same procedure, and the two measurements were separated by an interval of half an hour to allow for recovery. Statistical analysis was performed as described earlier (Section 2.1.5 ). 

## 3. Results

### 3.1. The Hyperalgesia Model

**Figure 4:**
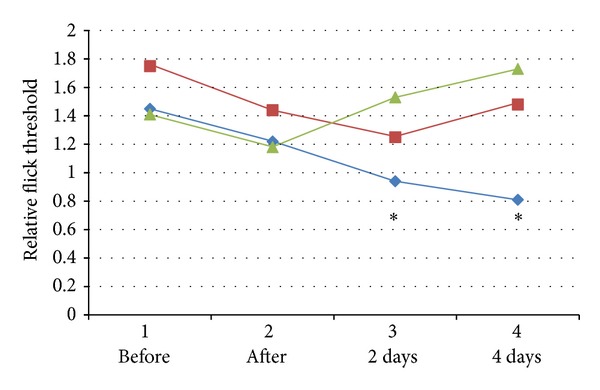
The changes in relative flick threshold after injecting hydrogel into LHRP (blue line), injecting saline into LHRP (red line), and injecting hydrogel into HHRP (green line). *P < 0.05 compared with RFT before the blocking.

The average RFTs before injection, immediately after injection, two days after, and four days after the injection are shown in Table 1 and Figure 4 for the three groups. 

One-way ANOVA analysis showed a significant decrease of RFT in the LHRP group (*F*_[3,76]_ = 7.977, *P* = 0.000). No significant differences were found between the group of saline injection (*F*_[3,76]_ = 1.096, *P* = 0.356) and the group of HHRP injection (*F*_[3.76]_ = 1.232, *P* = 0.304). In the LHRP group, there were no significant differences between the RFT before and immediately after the injection, while the RFT significantly decreased after 2 days and 4 days compared with the RFT before and immediately after the injection. There was no significant difference in the RFT at 2 days and 4 days after the injection. 

### 3.2. The Change of Hydraulic Resistance after Acupuncture in Pigs

**Figure 5:**
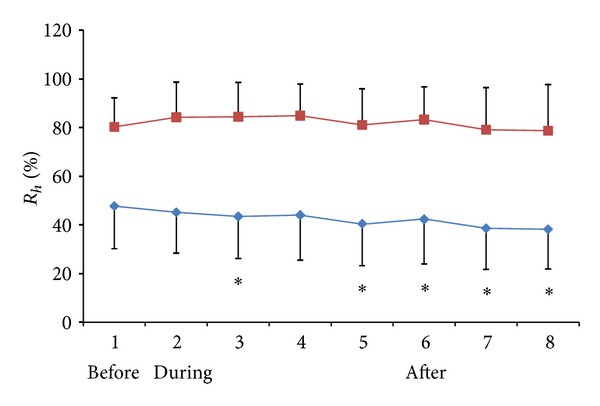
The changes of HR on LHRP (blue line) and HHRP (red line) before, during, and after acupuncture in pigs. *P < 0.05 compared with the HR before acupuncture.

The effects of acupuncture on HR were observed on 13 low impedance meridian lines. Relative HR (*R*_*h*_%) was calculated by the formula *R*_*h*_% = (*R*_*h*_(*p*) − *R*_*h*_(0))/(*R*_*h*_(*∞*) − *R*_*h*_(0)). *R*_*h*_(*∞*) is the resistance when the measuring needle is totally closed and *R*_*h*_(0) is the resistance when the measuring needle is totally open. The *R*_*h*_% following each time interval in the LHRP and HHRP groups is shown in Table 2 and Figure 5. 

One-way ANOVA analysis showed no significant decrease of HR when measured at the designated time intervals in either of the two control groups (*F*_[7,96]_ = 0.443, *P* = 0.873 in LHRP; *F*_[7,96]_ = 0.368, *P* = 0.919 in HHRP). However, at several time points (4′ and 8′ during the acupuncture and 0′, 5′, and 10′ after withdrawing the needle), *R*_*h*_% was significantly lower than before the acupuncture in the LHRP group, implying a reduction of HR on the LHRC along meridians through acupuncture.

## 4. Discussion

### 4.1. The Hyperalgesia Model of Blocking Meridian Channels

Pain is attributed to stasis or stagnation in meridian channels in classical acupuncture theory, and acupuncture was indicated to dredge the channels in order to relieve pain. This important idea about the mechanism of action of acupuncture had been ignored by modern doctors because neither the true nature of the meridian channels nor the condition of stasis in the meridian channels has been well understood by modern people. Our previous study implied that the true nature of meridian channels is at least partially related to interstitial fluid flow under the conditions of low hydraulic resistance channel along the meridians [15]. This new discovery made it possible to design a study to gauge whether pain appears when the channel is blocked and to determine if acupuncture can alter the condition of the channel. This corresponds quite well to the classic theory.

Polyacrylamide hydrogel was firstly used to treat asynodia in 1983. It has been widely used in cosmetic surgery and breast enhancement, but its safety has not been considered from the perspective of meridian channels, and there were complication reports on long-term use of the material [18]. Polyacrylamide hydrogel was chosen to create stasis in the meridian channels due to the fact that it is already being used as a biological filling material without causing obvious inflammation over a fourteen-month period [19]. The gel can increase the hydraulic resistance in the meridian channels, stopping the interstitial flow due to the high hydraulic resistance as reported by Scott et al. and Coleman et al. [20, 21]. Our previous study showed that the injection of hydrogel can efficiently block the transmission of interstitial fluid pressure waves when the amount of injection was equal or beyond 0.5 mL [22]. Therefore, 0.5 mL gel was given because this is adequate for blocking but is the least amount possible to minimize the disturbance to the animal. 

As pigs cannot report pain themselves, the tail-flick threshold and ear-flick threshold were used to approximately represent the pain threshold which is often applied in animal experiments of pain. Therefore, our model is not exactly a pain model but a hyperalgesia model which involves the same mechanism of pain. The relative flick threshold was used to diminish the possible influence of anaesthesia and to make the results more reliable. As it was rather difficult to test a large number of pigs, unlike rats and rabbits, samples were obtained from different places on each pig and the data was processed using the Wilcoxon Signed Ranks Test.

The results showed a slight decrease of RFT in all three groups immediately after the injection, although it was not significant. The reason for this may be that the injections themselves made pigs more sensitive to the hot stimulation which is in fact not related to the subject injected. Nevertheless, only the injection of hydrogel at LHRP along the channel appeared to produce a progressive hyperalgesia to the stimulation two days after the injection, while no such change occurred in the other two control groups. The results imply that hyperalgesia occurred where the LHRC along meridians was blocked. This could be understood by an accumulation of metabolite containing many H^+^ chemicals on LHRC which induces a depolarization of nerve terminals and therefore the process required some time to occur.

How does interstitial fluid flow and what is the function of the flow? Modern physiology focuses on blood flow but few studies have been carried out on interstitial flow. Aukland and Reed discussed the function of interstitial flow on balancing extracellular fluid and preventing edema in 1993 [23]. Swartz and Fleury pointed out in 2007 that interstitial flow affects more than just cell nourishment: it can, for example, induce blood and lymphatic capillary morphogenesis in vitro and lymphatic regeneration in vivo, maintain the functional activity of chondrocytes and osteocytes, drive fibroblast differentiation, and induce cytokine production by smooth muscle cells [24]. If such an important flow is stopped, a pathological state will develop resulting in not only pain but also diminished physiological functions in many areas.

### 4.2. The Mechanism of Dredging Meridian Channels through Acupuncture

If we accept that the true nature of the meridian channels can be understood as low hydraulic resistance channels and the stasis in these channels can be understood as an increase of HR in these channels, then the classic technique of “dredging meridian channels” can be understood as a literal description. From this perspective, acupuncture can be understood as decreasing HR in the meridian channels and restoring the interstitial flow to eliminate wastes from the tissue. Our experiment on the pigs showed a significant decrease of HR in LHRC during and after the acupuncture but no obvious change on the control point outside the channel. The idea of dredging meridian channels as formulated in the *Ling Shu *section of the *Yellow Emperor's Inner Canon* was intended as a general principle for both medical treatment and preventative healthcare. The latter results, taken in combination with the induction of hyperalgesia we have found in the first experiment, suggest a comprehensive explanation for the curative effects of acupuncture for treating chronic pain. Specifically, acupuncture reduces the resistance in the meridian channels and improves interstitial fluid flow, aiding in the elimination of wastes and improving the local internal milieu which eventually leads to the recovery of the normal function of nerve terminals and the reduction of pain. This process also has positive effects on motor and endocrine functions.

**Figure 6:**
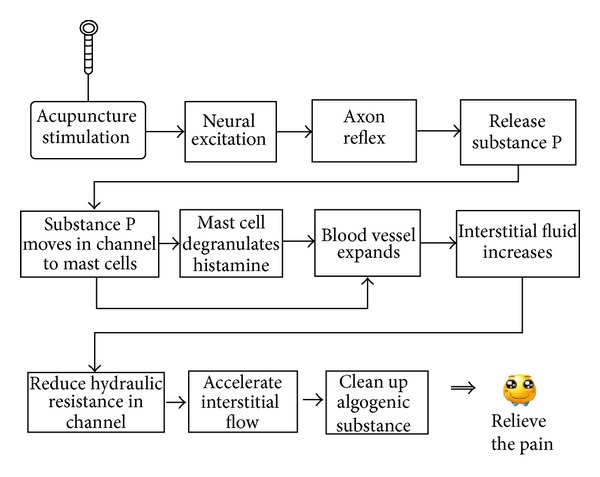
A mechanism of action of acupuncture in treating chronic pain.

The exact mechanism of how acupuncture decreases HR in the meridian channels requires further study in order to be answered. Two mechanisms are involved in the change. One is the pathway related to the blood vessel system. When a needle is inserted into an acupoint, the nerve terminal which usually consists of A_*δ*_ or C fibers is excited and sends a neural pulse signal to the central nerve system. Then, an axon reflex develops which leads to the release of substance P (SP) and other chemicals into the interstitial fluid around the needle. SP and other signals diffuse and migrate to mast cells nearby through interstitial flow along meridian channels. Mast cells then degranulate and release histamine which will continue to move along meridian channels. SP and histamine can make surrounding blood vessels expand and become more permeable allowing more interstitial fluid to flow outside the vessel. This accelerates the flow along meridian channels and reduces the hydraulic resistance along the channels. The lower resistance further facilitates the interstitial flow which can eliminate algogenic substances to relieve pain (Figure 6). 

The release of SP during acupuncture and its influence on neurons across segments have been proven experimentally by Zhang et al. [25, 26]. The degranulation of histamine from mast cells was also proven to play an important role in the analgesic effects of acupuncture [27]. The migration of neurotransmitters and other chemicals in extracellular space was named “volume transmission (VT)” by Agnati et al. in 1986 [28], and VT in peripheral tissue along meridians was discussed recently as an explanation for an important meridian phenomenon: propagated sensation along meridian [29]. The increase of blood perfusion and an increase of interstitial fluid represented by the decrease of electric impedance have been observed by Zhang et al. [30, 31]. The new evidence in this paper seems to indicate that the role of blood is essential in the mechanism of action of acupuncture.

The other pathway might be through the kinetic system. Due to biophysical principles, the activity of muscle will influence the interstitial flow tremendously. A rhythmic contraction can enhance the interstitial flow while a tetanic contraction or atrophy will diminish the flow. Acupuncture can help the muscles return to a normal state due to the kinetic reflex effected through the muscle spindle reflex or the tendon reflex. This regulation can even act on a remote place along meridians through *α* motor neuron chains in the spinal cord, as has been found by Xie et al. in 1995 [32]. Myoelectricity during acupuncture has been observed by several researchers [33, 34], and an increase of HR when a tetanic contraction developed has been found in our lab recently [35]. Direct evidence of improving interstitial flow is still absent although there is indirect evidence that acupuncture can accelerate the migration of isotope ^99m^TcO_4_^−^ along meridians [36]. More experiments should be undertaken to prove the kinetic mechanism of acupuncture.

Xuan Ze-ren in China developed his theory of aseptic inflammation as the cause of some chronic pain such as lumbar disc herniation [37]. Xuan has applied the special treatment of soft tissue lysis on many cases instead of classic resection of lumbar disc herniation and has reported good results [38, 39]. Acupuncture can facilitate the interstitial flow by reducing the resistance in the channels. Other treatments in TCM may have similar mechanisms. For example, moxibustion enhances the interstitial flow by expanding blood vessels; massage and cupping enhance the interstitial flow by changing interstitial fluid pressure; meridian-sinews release therapy can release the extra interstitial fluid which contains inflammatory substance and reduce abnormally high fluid pressure. According to the terminology of traditional Chinese medicine, acupuncture “dredges” meridian channels, making *qi* and *blood* flow more efficiently so that pain is eliminated. The concept of lowering hydraulic resistance is quite consistent with this classical idea and the relief of pain can be logically conceptualized as the elimination of algogenic substances through stronger interstitial flow induced by lowering the resistance in meridian channels.

### 4.3. The Relationship between Acupuncture Effects, Meridian Channels, and the Nervous System

Longhurst put forward a definition for meridian channels, stating that it is an entity that, when stimulated by acupuncture, can result in clinical improvement [40]. The mechanism of action of acupuncture on peripheral tissue, in which LHRC plays the key role, fits this definition quite well and does not deny the role of the nervous system. Longhurst also posited out that the peripheral and central nervous systems can now be considered to be the most rational basis for defining meridian channels [40], a view which is held by many other scholars. However, an entity which is related to an acupuncture effect does not mean it is the meridian channel. To determine whether an entity is the meridian channel, several criteria need to be met, including distribution patterns, the function of nourishing tissue, “smoothing joints,” regulating organs and resisting evil. These should be taken into account apart from the acupuncture effect. More essentially, the characteristics of a channel as something that restricts material or energy, runs along a specific route and is in an opened or closed state should be elucidated. We know that the main function of nerves is transporting neural signals, and that they are not responsible for diminishing inflammation, resisting bacteria, healing tissue, and so on. Also, the transportation of neural signals is very fast and difficult to be stopped by mechanical pressure which is not similar to the characteristics of propagated sensation along meridians. Neural excitation is indeed the first step in the cascade of events of the acupuncture effect. But to get a real long-term effect, a permanent change, neural excitation is not enough. There must be a chain of actions to finish the effect. Neural excitation is also not the only pathway for acupuncture effects. Manipulation in acupuncture is an important step to get a good effect but its contribution to neural excitation is small. Langevin et al. found a winding of collagen around acupuncture needle after a manipulation of bidirectional rotation [41]. Ding further found a positive relationship between the twist of collagen, degranulation of mast cell, and acupuncture analgesia [42]. Degranulation of mast cell is a middle step which can be induced not only by axon reflex of neural excitation but also by the shearing motion of collagen during acupuncture manipulation. There are multipathways for the effects of acupuncture. A meridian channel entity should be defined not only by the effects of acupuncture but also should also include other aspects.

## 5. Conclusion

Blocking meridian channels by injecting hydrogel causes a hyperalgesic state. The mechanism of action of acupuncture in treating chronic pain can be understood as a reduction of hydraulic resistance in meridian channels which accelerates interstitial flow, eliminates algogenic substances, and ultimately relieves pain. 

## Figures and Tables

**Table 1:** The relative flick threshold (RFT) before, immediately after, two days, and four days after the injections in three groups: hydrogel injected into LHRP, saline injected into LHRP, and hydrogel injected into HHRP.

Groups	Before	After	2 days	4 days
LHRP	1.45 ± 0.62	1.22 ± 0.46	0.94 ± 0.31^∗Δ^	0.81 ± 0.29^∗Δ^
Saline	1.75 ± 1.18	1.44 ± 0.74	1.25 ± 0.60	1.48 ± 0.86
HHRP	1.41 ± 0.95	1.18 ± 0.62	1.53 ± 0.85	1.73 ± 1.02

				Means ± SD, *n* = 20

*P < 0.05 compared with the value before the injection.

^Δ^ *P* < 0.05 compared with the value immediately after the injection.

**Table 2:** Changes in HR on LHRP and HHRP due to acupuncture needling.

Groups	Before	1′ during	4′	5′	8′	0′ after	5′	10′
LHRP	47.7 ± 17.7	45.2 ± 16.7	43.5 ± 17.2*	44.0 ± 18.5	40.5 ± 17.2*	42.3 ± 18.4*	38.6 ± 16.8*	38.2 ± 16.4*
HHRP	80.3 ± 12.0	84.2 ± 14.5	84.4 ± 14.1	84.9 ± 13.0*	81.1 ± 14.8	83.3 ± 13.5	79.1 ± 17.3	78.7 ± 18.9

								Means ± SD, *n* = 13

*P < 0.05 compared with the HR before acupuncture.
